# Correction: The Cyprus Institute of Neurology and Genetics, an emerging paradigm of a gender egalitarian organisation

**DOI:** 10.1371/journal.pone.0316685

**Published:** 2024-12-27

**Authors:** Stavroulla Xenophontos, Margarita Zachariou, Pavlos Polycarpou, Elena Ioannidou, Vera Kazandjian, Maria Lagou, Anna Michaelidou, George M. Spyrou, Marios A. Cariolou, Leonidas Phylactou

Fig 1B is incorrect. The authors have provided a corrected Fig here.

**Fig 1:**
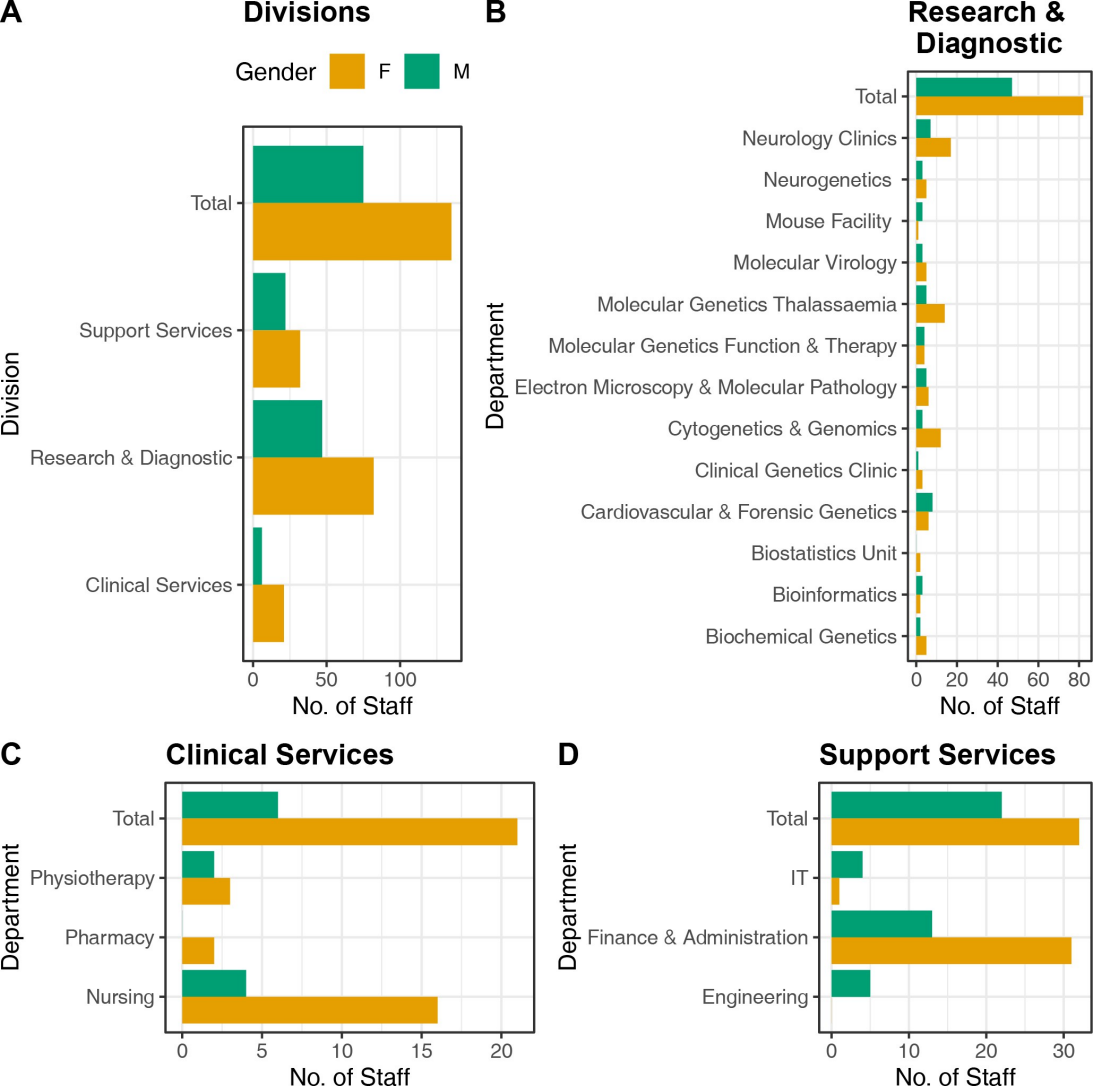
Gender distribution of employees in the CING. **A.** Histogram plots of the gender distribution of employees in the three divisions (Clinical Services, Research & Diagnostic and Support Services) in the CING. **B.** Histogram plots of the gender distribution of employees in the CING Departments (Clinics, Laboratories and Facilities). **C.** Histogram plots of the gender distribution of employees in the Clinical Services departments. **D.** Histogram plots of the gender distribution of employees in the Support Services departments of the CING.
