# Supplementary material for: The Cyprus Institute of Neurology and Genetics, an emerging paradigm of a gender egalitarian organisation
Source: PLoS One. 2022 Sep 15;17(9):e0274356. doi: 10.1371/journal.pone.0274356 (PMC9477314; doi:10.1371/journal.pone.0274356)
Supplement: S1 Table — (PDF) [file pone.0274356.s001.pdf]

Table S1 – Gender Distribution in the CING Divisions.

CING Division Category	Males	Females	Total
**Clinical Services**	6	21	27
**Research and Diagnostic**	47	82	129
**Support Services**	22	32	54
**Total**	75	135	210
