# Supplementary material for: The Cyprus Institute of Neurology and Genetics, an emerging paradigm of a gender egalitarian organisation
Source: PLoS One. 2022 Sep 15;17(9):e0274356. doi: 10.1371/journal.pone.0274356 (PMC9477314; doi:10.1371/journal.pone.0274356)
Supplement: S2 Table — (PDF) [file pone.0274356.s002.pdf]

Table S2: Gender Distribution in the CING Departments

Department/Facility	Males	Females	Total
**Biochemical Genetics**	2	5	7
**Bioinformatics**	3	2	5
**Biostatistics Unit**	0	2	2
**Cardiovascular & Forensic Genetics**	8	6	14
**Clinical Genetics Clinic**	1	3	4
**Cytogenetics and Genomics**	3	12	15
**Electron Microscopy & Molecular Pathology**	5	6	11
**Molecular Genetics Function & Therapy**	4	4	8
**Molecular Genetics Thalassaemia**	5	14	19
**Molecular Virology**	3	5	8
**Neurogenetics**	3	5	8
**Neurology Clinics**	7	17	24
**Mouse Facility**	3	1	4
**Total**	47	82	129
