# Supplementary material for: The Cyprus Institute of Neurology and Genetics, an emerging paradigm of a gender egalitarian organisation
Source: PLoS One. 2022 Sep 15;17(9):e0274356. doi: 10.1371/journal.pone.0274356 (PMC9477314; doi:10.1371/journal.pone.0274356)
Supplement: S3 Table — (PDF) [file pone.0274356.s003.pdf]

Table S3: Gender Distribution in the CING Clinical Services

Department/Facility	Males	Females	Total
**Nursing**	4	16	20
**Pharmacy**	0	2	2
**Physiotherapy**	2	3	5
**Total**	6	21	27
