# Supplementary material for: The Cyprus Institute of Neurology and Genetics, an emerging paradigm of a gender egalitarian organisation
Source: PLoS One. 2022 Sep 15;17(9):e0274356. doi: 10.1371/journal.pone.0274356 (PMC9477314; doi:10.1371/journal.pone.0274356)
Supplement: S4 Table — (PDF) [file pone.0274356.s004.pdf]

Table S4: Comparison of Gender Distribution in the CING Support Service Departments

Department	Males	Females	Total
**Finance & Administration**	13	31	44
**Engineering**	5	0	5
**IT**	4	1	5
**Total**	22	32	54
