# Supplementary material for: The Cyprus Institute of Neurology and Genetics, an emerging paradigm of a gender egalitarian organisation
Source: PLoS One. 2022 Sep 15;17(9):e0274356. doi: 10.1371/journal.pone.0274356 (PMC9477314; doi:10.1371/journal.pone.0274356)
Supplement: S5 Table — (PDF) [file pone.0274356.s005.pdf]

Table S5: Comparison of Ranking Positions between Males & Females

Position	Males	Females	Total
**Head**	7	7	14
**Head (Senior Scientist)**	4	5	9
**Head (Senior Clinician)**	3	2	5
**Senior Associate**	2	0	2
**Scientist/Clinician**	11	4	15
**Scientist**	9	3	12
**Clinician**	2	1	3
**Associate Scientist**	7	13	20
**SLSO**	3	3	6
**Post-doc**	3	9	12
**LSO**	13	41	54
**Lab Assistant**	1	5	6
