# Supplementary material for: The Cyprus Institute of Neurology and Genetics, an emerging paradigm of a gender egalitarian organisation
Source: PLoS One. 2022 Sep 15;17(9):e0274356. doi: 10.1371/journal.pone.0274356 (PMC9477314; doi:10.1371/journal.pone.0274356)
Supplement: S6 Table — (PDF) [file pone.0274356.s006.pdf]

Table S6 Qualifications PhD and MD Qualifications in the Research & Diagnostic Division

Qualifications	Males	Females	Total
**PhD**	27	43	70
**MD**	5	3	8
**PhD & MD**	1	0	1
