# Supplementary material for: The Cyprus Institute of Neurology and Genetics, an emerging paradigm of a gender egalitarian organisation
Source: PLoS One. 2022 Sep 15;17(9):e0274356. doi: 10.1371/journal.pone.0274356 (PMC9477314; doi:10.1371/journal.pone.0274356)
Supplement: S7 Table — (PDF) [file pone.0274356.s007.pdf]

Table S7: Comparison of Years of Service between Males & Females at 4 Year Intervals

Years of Service & Recruitment Interval at the CING	Males	Females	Total
**1-4 years (2017-2020)**	20	31	51
**5-8 years (2013-2016)**	10	13	23
**9-12 years (2009-2012)**	12	21	33
**13-16 years (2005-2008)**	6	16	22
**17-20 years (2001-2004)**	9	20	29
**21-24 years (1997-2000)**	6	11	17
**25-28 years (1993-1996)**	5	16	21
**29-32 years (1989-1992)**	7	7	14
**Total**	75	135	210
