# Supplementary material for: The Cyprus Institute of Neurology and Genetics, an emerging paradigm of a gender egalitarian organisation
Source: PLoS One. 2022 Sep 15;17(9):e0274356. doi: 10.1371/journal.pone.0274356 (PMC9477314; doi:10.1371/journal.pone.0274356)
Supplement: S8 Table — (PDF) [file pone.0274356.s008.pdf]

**Table S8: Postgraduate Student Successive Academic Year Student Gender**

| Academic Year | Males | Females | Total |
|---|---|---|---|
| **2012-2013** | 12 | 24 | 36 |
| **2013-2014** | 12 | 17 | 29 |
| **2014-2015** | 3 | 18 | 21 |
| **2015-2016** | 7 | 34 | 41 |
| **2016-2017** | 3 | 20 | 23 |
| **2017-2018** | 4 | 24 | 28 |
| **2018-2019** | 8 | 26 | 34 |
| **2019-2020** | 11 | 20 | 31 |
| **Total** | 60 | 183 | 243 |
